# Pleomorphic Adenoma-Like Tumor in the Kidney: Case Report of a Rare Mixed Tumor

**DOI:** 10.1155/crin/8959506

**Published:** 2025-11-12

**Authors:** Eric Y. Zhang, Haneen T. Salah, Danice K. Torman, Mukul K. Divatia, Haijun (Steve) Zhou

**Affiliations:** ^1^Department of Pathology and Genomic Medicine, Houston Methodist Hospital, Houston, Texas, USA; ^2^Department of Pathology, The University of Alabama at Birmingham, Birmingham, Alabama, USA; ^3^Department of Pathology, The University of Texas MD Anderson Cancer Center, Houston, Texas, USA; ^4^Department of Laboratory Medicine and Pathology, University of Washington, Seattle, Washington, USA; ^5^Weill Medical College of Cornell University, Houston, Texas, USA

**Keywords:** adenoma, kidney neoplasms, malignant, mixed tumor, neoplasm metastasis, pleomorphic, salivary gland neoplasm

## Abstract

**Background:**

Pleomorphic adenoma is the most common salivary gland tumor and is notably able to metastasize while displaying a benign-appearing morphological appearance, greatly resembling that of the primary tumor.

**Case Report:**

Here, we report a case of a 66-year-old male who presented with a 7-month history of a left upper renal mass. Microscopic examination revealed characteristics typical of pleomorphic adenoma, including chondromyxoid stroma and benign ductal and tubular cells. Consequently, a diagnosis of benign mixed tumor resembling a metastasizing pleomorphic adenoma (MPA) to the kidney was made.

**Conclusions:**

We report a rare instance of a tumor resembling pleomorphic adenoma with metastasis to the kidney, the 13^th^ reported case of kidney metastasis in the literature. This case is additionally the 2^nd^ case with metastases to the kidney and no known primary salivary gland tumor. We believe that this entity is most consistent with the histopathological findings reported in our case, despite the lack of a patient history of primary salivary gland tumor. We subsequently compare patient outcomes across all known cases of benign MPA to the kidney or PA-like tumors in the kidney with patient outcomes described in a 2015 study that analyzed all cases of MPA in the literature.

## 1. Background

Pleomorphic adenomas are the most common type of salivary gland tumors, accounting for ∼50% of these neoplasms. Grossly, pleomorphic adenomas appear as a well-circumscribed, encapsulated mass, and are marked histologically by epithelioid or myoepithelial cells within a morphologically diverse stromal component that may include chondromyxoid, fibrous, mucoid, or osseous elements [1].

Characteristic immunohistochemical features that can suggest the diagnosis of pleomorphic adenoma include diffuse and strong reactivity for cytokeratin immunohistochemical markers, especially in the epithelial cell component. Muscle, myoepithelial, or basal markers, including calponin, SMA, S100, p40, SOX10, and CK5/6, can also be positive [2]. Pleomorphic adenomas can harbor gene fusions involving *PLAG1* on 8q12 (seen in > 50% of cases) or *HMGA2* on 12q14.3 (seen in 10%–15% of cases). Fusion partner genes for *PLAG1* include *CTNNB1*, *FGFR1*, *LIFR*, *CHCHD7*, *TCEA1*, *NFIB*, and *BOC*, while fusion partner genes for *HMGA2* include *NFIB*, *WIF1*, *FHIT*, or *TMTC2* [2].

Though pleomorphic adenomas are well-known for their potential to undergo malignant transformation into variants such as carcinoma ex pleomorphic adenoma, benign-appearing metastasizing pleomorphic adenoma (MPA) is a rare but well-documented entity [1]. The most common sites of distant metastasis include bone, followed by the head, neck, and lungs [3]. Histologically and molecularly, benign MPA is indistinguishable from benign pleomorphic adenomas at a primary location, and there are no known histological or molecular features that can reliably predict metastasis.

MPAs typically arise in patients with a history of previously resected pleomorphic adenomas, often manifesting long after primary tumor resection. One study describes an average interval of 12.3 years from initial presentation to metastasis onset, which is extended to 16.9 years in cases with a history of local recurrence [4]. In addition, the same study identified that incomplete surgical resection of tumor margins significantly increases the risk of metastatic spread in pleomorphic adenomas [4].

## 2. Case Report

**Figure 1:**
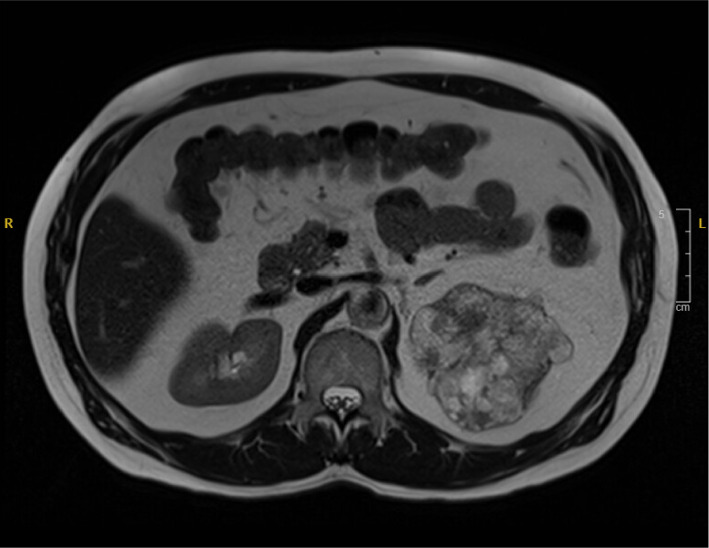
MRI shows an 8 cm, heterogeneously T_2_ hyperintense mass with internal enhancing nodular components.

**Figure 2:**
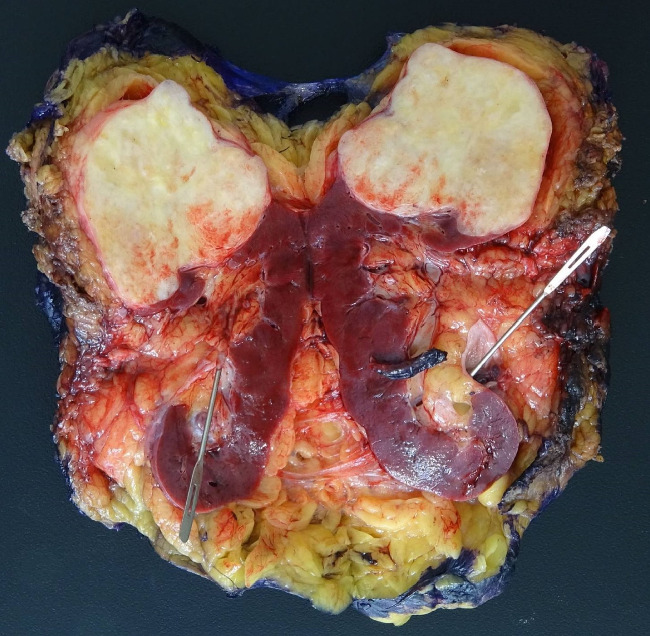
Photograph showing a 7.5 × 7 × 6 cm tan-white firm mass situated in the upper pole of the kidney. The cut surface of the mass is homogeneous and firm. The mass appears to be encapsulated and abuts the renal parenchyma and does not grossly appear to invade the renal pelvis.

**Figure 3:**
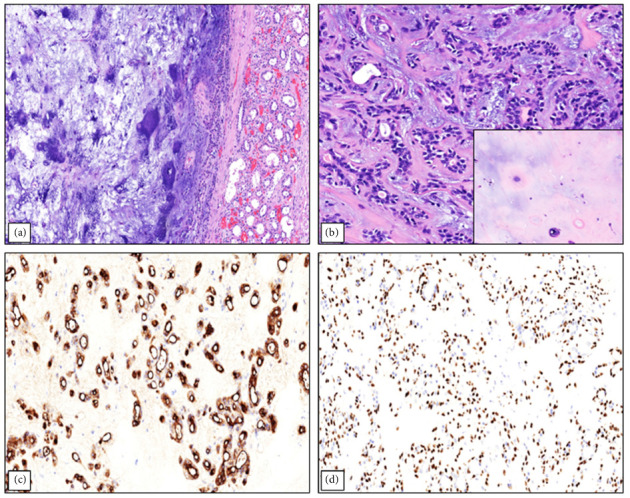
(a) Low-power microscopic image showing the encapsulated pleomorphic adenoma-like tumor in relation to adjacent normal kidney parenchyma (10X magnification). (b) Cellular areas of the tumor including tubular and ductal structures. Image insert: Hypocellular areas of the tumor showing a chondromyxoid stroma (20X magnification). (c) Immunohistochemistry showing positive staining for EMA in the luminal cells of the ducts and tubules (20X magnification). (d) Immunohistochemistry showing positive staining for p63 in the basal cells of the ducts and tubules (20X magnification).

A 66-year-old male with no significant medical history presented for a left radical nephrectomy at our institution, following an incidental discovery of an upper renal mass on a CT scan of the abdomen and pelvis approximately seven months earlier. The patient's physical examination was unremarkable, and no head and neck examination was performed due to low clinical suspicion of metastasis from the primary salivary gland tumor. MRI of the abdomen without contrast, performed 7 months prior to the surgery, showed an 8.5 × 8.0 × 6.0 cm heterogeneously T2 hyperintense left upper kidney mass with internal enhancing nodular components consistent with renal cell neoplasm. A second MRI of the abdomen without contrast, performed 3 months prior to the surgery, showed no changes in the size of the mass with no invasion of adjacent structures. No dedicated CT and MRI of the salivary glands were performed, for the same reason that a head and neck examination was not performed. During gross examination of the patient's resected left kidney, a tan-white, encapsulated mass measuring 8.4 × 8.1 × 6.8 cm corresponding to the mass identified on imaging was identified grossly in the upper pole of the kidney (Figure 1). The cut surface of the mass was homogeneous and firm, with no gross areas of hemorrhage or friability (Figure 2). Microscopic examination of the specimen revealed features resembling those of pleomorphic adenoma, including (Figure 3(a)) chondromyxoid stroma and areas of benign tubular and ductal cells (Figure 3(b)). Our complete immunohistochemical workup showed positivity for EMA (Figure 3(c)), GATA3, and CD117 in ductal and tubular cells; PAX8 positivity in renal tubular epithelium; and p63 (Figure 3(d)), SOX10, and S100 positivity in basal cells. Ki67 staining indicated low proliferative activity (1%-2%). Immunohistochemistry for WT1 and BRAF V600E were not performed. Despite the known utility of *PLAG1* immunohistochemical testing in the diagnosis of pleomorphic adenoma, we elected to not perform this test because the morphological and immunohistochemical features of the tumor were so strongly consistent with pleomorphic adenoma. At a follow-up visit by urology, 3 weeks postoperation, the patient was in good condition and showed no complications or signs of recurrence. We acknowledge the limitations of such short-term follow-up in detecting complications, but 2 years and 2 months postoperation, the patient still has no clinical evidence of complications or recurrence, indicating that the patient's immediate postoperative follow-up was sufficient. Notably, no history of previous pleomorphic adenoma was identified in the patient's medical records, nor did the patient's primary care provider know of any history of salivary gland neoplasms.

## 3. Discussion

Only 12 cases of MPA to the kidney have been reported in the literature [3–11] (summarized in Table 1). Metastasectomy is currently the only therapeutic modality known to increase survival in patients with metastatic pleomorphic adenoma [4].

Prognostic factors and laboratory findings that can predict the metastatic dissemination of pleomorphic adenoma are limited. One proposed mechanism for the development of metastatic pleomorphic adenoma is incomplete surgical resection. Another involves hematogenous dissemination of residual tumor cells from the primary tumor to other organs, consistent with observations that a history of incomplete surgery for the primary tumor increases the risk of metastasis. In addition, previous recurrences of the primary tumor are known to increase the risk of metastasis [4].

Apart from pleomorphic adenoma, our differential diagnosis included other biphasic solid renal tumors, such as mixed epithelial and stromal tumor (nephroma), metanephric adenofibroma, and mucinous tubular spindle cell carcinoma (Table 2). While pleomorphic adenoma and metanephric adenofibroma can be differentiated based on a history of prior salivary gland tumor, metanephric adenofibroma is associated with strong nuclear immunoreactivity for WT1 and BRAF V600E in the epithelial component. Morphologically, pleomorphic adenoma differs from metanephric adenofibroma in that it tends to have more prominent myxoid stroma and the presence of myoepithelial cells [15, 16]. Though MPA and papillary adenoma both exhibit similar microscopic characteristics, the two can be differentiated by the more prominent stromal component in MPA, compared to the more epithelioid appearance of papillary adenoma, in addition to positivity for CK7, AMACR and vimentin in papillary adenoma, which are all negative in pleomorphic adenoma [17]. Mucinous tubular and spindle cell carcinoma is also a biphasic tumor but can be distinguished by its prominent tubular component with extensive branching and interconnecting tubular architecture and is PAX8-, EMA-, CK7-, and AMACR-positive [11]. Finally, mixed epithelial and stromal tumor is a cystic mass and thus differs markedly from the solid mass of MPA in gross appearance and shows a markedly different immunohistochemical profile: mixed epithelial and stromal tumor is notably positive for ER and PR, smooth muscle actin, and CD10 (especially around epithelial elements) [12, 15, 17].

Despite the efficacy of PLAG1 and HMGA2 testing in the diagnosis of pleomorphic adenoma, especially in an unusual site such as the kidney, in the absence of a prior history of salivary gland neoplasm, we did not perform these stains as we do not carry stains for PLAG1 and HMGA2. Generally, at our institution, we can diagnose even difficult cases of pleomorphic adenoma based on morphology, and given that this case showed classic features of pleomorphic adenoma (although in an unusual location), we believed that we could make an accurate diagnosis without needing to use PLAG1 and HMGA2 immunohistochemistry. We note that during the initial clinical evaluation, no detailed head and neck examination or salivary gland imaging was performed due to low clinical likelihood of metastasis from a primary salivary gland tumor. We acknowledge this as a limitation, as diagnosis of metastasis requires exclusion of a primary tumor at the site of involvement, and no clinical or imaging evidence is available to prove the existence of a primary salivary gland tumor.

With respect to quality of life (QOL) aspects in patients with MPA, one significant limitation is that many of the case reports that discuss this entity do not document long-term follow-up. A 2015 review by Knight et al. of all extant cases of MPA focuses on survival rates in patients with MPA as the primary QOL metric as a means of dealing with this limitation, although the poor documentation of long-term follow-up limits the conclusions that can be drawn from this study. His study found that 51 out of 81 cases studied (62.9%) reported survival or disease-free periods, and of these cases, 9 out of the 51 cases (17.6%) reported patient death due to MPA, 11 of the 51 cases reported a disease-free period for periods ranging from 12 to 84 months, and 40 of the 51 cases reported a disease-free period but did not report further follow-up [18].

In our study, we found that 9 out of the 13 case reports with the final diagnosis of MPA (69.2%) included information on survival or disease-free periods (our case and both of the cases described by Wenig et al., Ebbing et al., Mohan et al., Lobo et al., Xiao et al., Koyama et al., and Cherian et al.) [3, 8–11, 13, 19]. Two case reports out of the nine with documented disease-free or survival periods describe the patient dying due to direct complications of benign MPA (Xiao et al. and Cherian et al.) (22.2% of all cases). Two case reports of the nine (22.2%) describe survival with disease from 12 months to 84 months after treatment for the MPA (Xiao et al. and Koyama et al.) [8, 10, 13]. Four of the nine case reports (44.4%) describe disease-free periods with loss to follow-up (one case by Wenig et al., who presented with primary salivary gland tumor at age 34 and metastasis at age 65, Lobo et al., Ebbing et al., and Mohan et al.) [3, 9, 11, 19].

In comparing the cases discussed by our study to the cases discussed by Knight et al., most cases reported either survival or disease-free states in patients after treatment for benign MPA (69.2% in our study, compared to 62.9% in the study by Knight et al.). Two of our case reports with documented survival or disease-free states after treatment reported death directly from MPA (22.2% of all cases), compared to 17.6% of the cases described by Knight et al. (9/51 cases) [10, 13, 18]. Two of our case reports describe survival with disease for time periods ranging from 12 to 84 months (22.2% of all cases), compared to 11 case reports described by Knight et al. (21.6% of 51 total cases) [8, 10, 18]. Four of our nine case reports describe disease-free periods with loss to follow-up (44.4%), compared to 41 of the 51 cases described by Knight et al. (78.4% of all cases) [3, 9, 11, 18, 19]. This analysis suggests that overall prognosis in terms of patient survival does not differ significantly between benign MPA in general compared to MPA with metastases to the kidney, although definite statements are difficult to make due to discrepancies in the documentation of survival across all case reports.

## 4. Conclusions

Despite the rarity of MPA, the possibility of future metastatic disease should be considered in the surgical management of all cases of pleomorphic adenomas. Positive surgical margins significantly increase the risk of MPA. Therefore, meticulous surgical excision of primary pleomorphic adenoma tumors is crucial, as remaining tumor cells can serve as a nidus for future metastasis [4].

Effective communication between the referring clinician and the pathologist is essential for diagnosis, particularly considering the rarity of metastasis. Finally, understanding the unambiguous utility of metastasectomy in treating metastatic pleomorphic adenoma is critical [20, 21]. Operative treatment should be preferred over chemotherapy and radiation therapy for managing pleomorphic adenoma [4].

## Figures and Tables

**Table 1:** A summary of demographic, clinical, pathological, immunohistochemical and molecular features of reported cases of MPA to the kidney.

#	Study	Age (years)/sex	Maximum tumor dimension (cm)	Histologic appearance	Patient prognosis	Time between initial presentation and identification of metastatic disease (years)	Site of primary tumor	Available IHC and molecular workup
1/2	Wenig, 1992 [10]	51/F, 34/F	11/3	“Duct-like structures composed of a double layer of cells with an inner single layer of luminal cells surrounded by more basophilic-appearing spindle-shaped myoepithelial cells”/“glandular structures set in a myxoid tissue, with a portion of the metastatic mixed tumor showing cytologically bland ductal structures associated with a myxoid tissue”	Patient died without disease 1 year after nephrectomy/patient alive without disease 2 years after metastasis, status postnephrectomy	27/31	Parotid (laterality unspecified)/parotid (laterality unspecified)	N/A/N/A
3	Cherian, 1992 [12]	30/F	5	“Pleomorphic adenoma with carcinomatous areas, which showed a poorly differentiated adeno-pattern composed of cells having eosinophilic cytoplasm and pleomorphic vesicular nuclei with prominent nucleoli and mitotic figures, and myxoid stroma that showed spindle-shaped sarcomatous cells”	Patient developed multiple metastases to the right kidney, left submandibular lymph node, and the right trunk and fingers, and died at home 6 months later	2	Left parotid gland	N/A
5	Xiao, 2008 [9]	72/F	2.8	“Mixture of epithelial and mesenchymal-like elements, with sheets and duct-like, microcystic structures with foci of squamous differentiation”	Patient developed multiple bone metastases 34 months after nephrectomy and eventually died	26	Left parotid gland	“The tumor cells expressed p63, S100 protein, P53, GFAP, and vimentin. The tumor cells stained negatively for smooth muscle actin and CD10 and the MIB-1 (Ki67) labeling index was low (less than 1%)”
6	Bhutta, 2009 [5]	76/F	N/A	“Network of minimally pleomorphic cells with myoepithelial appearance, and infiltrative irregular tumor edge with invasion into vascular connective tissue stroma, and production of extracellular mucoid matrix”	N/A	13	Left parotid gland	“Staining with Ki67 showed cells in all stages of the mitotic cycle”
7	Ebbing, 2009 [3]	49/F	Two tumor foci: 0.20 and 0.50 cm	“Mixed cell composition with mesenchymal elements, demonstrating chondroid differentiation embedded in myxoid matrix, as well as epithelial components consisting of ductal structures without atypia”	Patient is status post nephrectomy and had no complications as of the publication of the article	29	Left parotid gland	“Immunohistochemical studies showed that the epithelial component stained positive for pan-cytokeratin AE1/AE3 with partial expression of the myoepithelial markers p63 and a very low Ki67 proliferation rate”
8	Vivian, 2012 [13]	40/F	2.4	“Abundant metachromatic fibrillary stromal matrix, cohesive epithelial cells, and myoepithelial cells”	N/A	16	Right parotid gland	N/A
9	Kessoku, 2014 [6]	64/F	N/A	N/A	N/A	N/A	Left parotid gland	“The Ki67 index in this case was 5.2% in the bone metastasis, 18.2% in the kidney metastasis, 5.5% in the lymph node metastasis, and 8.7% in the lung metastasis”
10	Koyama, 2014 [7]	63/F	N/A	“Myxoid epithelial tumor, (+) for pan-CK AE1/3 in epithelial cells and for p63 in myoepithelial cells”	Patient developed metastatic disease to the cavernous sinus, bone, lung, mediastinum and kidney	28	Left parotid gland	“Immunohistochemical staining was positive for pan-cytokeratin AE1/3 in the epithelial cells and p63 in the myoepithelial cells”
11	Mohan, 2018 [8]	38/F	N/A	“Benign biphasic neoplasm mimicking PA”	Patient is asymptomatic on follow-up	21	Parotid gland (laterality unspecified)	“Immunohistochemically, tumor cells expressed CK7 (epithelial cells), p63 (myoepithelial cells), S100 (myoepithelial cells and cells of chondromyxoid matrix) and very low MIB-1 (Ki67)–labeling index (< 1%)”
12	Lobo, 2023 [14]	43/F	8	“Triphasic appearance, composed of 2 populations of cells (epithelial and myoepithelial) and stroma”	Disease-free at 24 months after kidney surgery	31	Salivary gland (unspecified laterality or specific gland location)	“There were areas with tubular/glandular structures, strongly positive for CK7, CK19, and for pan-cytokeratin, and surrounding smaller, basaloid and spindle myoepithelial cells, strongly positive for S100, GFAP, SMA, calponin, and p63 but weakly positive for pan-cytokeratin. Tumor cells showed diffuse nuclear positivity for PLAG1; NGS detected CTNNB1::PLAG1 (split read 60.21%) and CHCHD7::PLAG1 (split read 18.48%) fusion, suggestive for a PA in the kidney”
13	Current case	66/M	8.4	“Well-circumscribed mass composed of abundant chondromyxoid stroma and benign tubular and ductal cells”	Disease-free at 26 months after kidney surgery	N/A	N/A	“Immunostaining demonstrated positivity for EMA (Figure 3), GATA3, and CD117 in ductal and tubular cells, and p63 (Figure 3), SOX10, and S100 in basal cells. Ki67 staining indicated low proliferative activity (1%-2%)”

*Note:* M: male, F: female.

Abbreviation: N/A, not available.

**Table 2:** A summary of additional entities on the differential diagnosis of mixed tumor in the kidney, with demographic, clinical, histological, and immunohistochemical features of these entities.

Diagnosis	Demographic and clinical features	Histological features	Immunohistochemistry (IHC)
Mixed epithelial and stromal tumor (MEST) [22]	Primarily seen in middle-aged women, often with a history of hormone exposure (mean age of diagnosis: 52 years)	Biphasic tumor with cystic epithelial components and a spindle cell stromal component resembling ovarian stroma	Epithelial component: positive for cytokeratin (e.g., CK7). Stromal component: positive for estrogen receptor (ER), progesterone receptor (PR), CD10, and smooth muscle actin (SMA)
Metanephric adenofibroma [18]	Typically seen in children and young adults (age range: 5 months–36 years)	Biphasic lesion with epithelial tubules and glands embedded in a fibrous stroma	Epithelial component: positive for WT1 and BRAF V600E. Stromal component: positive for vimentin and CD34
Mucinous tubular and spindle cell carcinoma [23]	Typically seen in middle-aged adults (median age: 50–60 years with female predominance)	Polymorphic, malignant renal cell neoplasm composed of bland anastomosing tubules, spindle cell areas, and myxoid stroma/extracellular mucin	Positive for PAX8, EMA, and CK7
Papillary adenoma [24]	More common in men with an increasing incidence with increasing age	Benign unencapsulated renal epithelial neoplasm characterized by papillary, tubular, or tubulopapillary architecture with low-grade nuclei and a diameter ≤ 15 mm	Positive for CK7, AMACR, and vimentin
